# Two New *Beggiatoa* Species Inhabiting Marine Mangrove Sediments in the Caribbean

**DOI:** 10.1371/journal.pone.0117832

**Published:** 2015-02-17

**Authors:** Maïtena R. N. Jean, Silvina Gonzalez-Rizzo, Pauline Gauffre-Autelin, Sabine K. Lengger, Stefan Schouten, Olivier Gros

**Affiliations:** 1 Institut de Biologie Paris-Seine, UMR 7138—Evolution Paris-Seine, Equipe Biologie de la Mangrove, Université des Antilles et de la Guyane, UFR des Sciences Exactes et Naturelles, Département de Biologie, BP 592. 97159 Pointe-à-Pitre cedex, Guadeloupe, France; 2 Department of Marine Organic Biogeochemistry, Royal NIOZ Netherlands Institute for Sea Research, P. O. Box 59, 1790AB Den Burg, Texel, The Netherlands; 3 Centre Commun de Caractérisation des Matériaux des Antilles Guyane, UFR des Sciences Exactes et Naturelles, Université des Antilles et de la Guyane, BP 592–97 159 Pointe-à-Pitre, Guadeloupe, French West Indies; U.S. Geological Survey, UNITED STATES

## Abstract

Beggiatoaceae, giant sulphur-oxidizing bacteria, are well known to occur in cold and temperate waters, as well as hydrothermal vents, where they form dense mats on the floor. However, they have never been described in tropical marine mangroves. Here, we describe two new species of benthic Beggiatoaceae colonizing a marine mangrove adjacent to mangrove roots. We combined phylogenetic and lipid analysis with electron microscopy in order to describe these organisms. Furthermore, oxygen and sulphide measurements *in* and *ex situ* were performed in a mesocosm to characterize their environment. Based on this, two new species, *Candidatus* Maribeggiatoa sp. and *Candidatus* Isobeggiatoa sp. inhabiting tropical marine mangroves in Guadeloupe were identified. The species identified as *Candidatus* Maribeggiatoa group suggests that this genus could harbour a third cluster with organisms ranging from 60 to 120 μm in diameter. This is also the first description of an *Isobeggiatoa* species outside of Arctic and temperate waters. The multiphasic approach also gives information about the environment and indications for the metabolism of these bacteria. Our study shows the widespread occurrence of members of Beggiatoaceae family and provides new insight in their potential role in shallow-water marine sulphide-rich environments such as mangroves.

## Introduction

*Beggiatoa* spp. are multicellular, filamentous colorless bacteria. Since their discovery by Vaucher in 1803, they are considered among the largest sulphur-oxidizing bacteria in nature [1]. Members of this genus are widespread in marine and freshwater environments. They often form mats on strongly reduced, organic or hydrocarbon-rich porous sediments, with sufficient interstitial space for motility [2]. Members of the Beggiatoaceae usually move by gliding motility [3] in order to respond to chemical signals [4]. They grow at the oxic/anoxic interface. They are usually found at the surface or within the top few centimeters of sulphide-rich sediments.

In marine environments, *Beggiatoa* spp. occur in different benthic habitats including hydrothermal vents [5], decomposing organic debris [6] and cold seeps [7]. In these environments, sulphide could be produced by fluid diffusion from geological sources (e.g. hydrothermal vents) or could also result from biological activity of the sulphate-reducing bacteria (SBR) metabolizing sulphate to sulphur [2,8]. Thus, *Beggiatoa* spp. are encountered from deep to coastal waters, and from cold to tropical waters [3,9,10,11]. In tropical environments, they often live together with phototrophic organisms such as cyanobacteria or micro-algae [9].

Marine mangroves are well known to be sulphide-rich environments [12]. Several sulphur-oxidizing bacteria were identified in these habitats, as free-living bacteria or as symbionts associated either with archaea [13], protists [12,14,15,16] or metazoans [16,17,18]. *Beggiatoa* mats, as well as mats of cyanobacteria, have already been observed in mangrove soils [19,20]. However, to our knowledge, no molecular characterizations of tropical strains of Beggiatoaceae from marine mangroves have ever been described.

*Beggiatoa* spp. are chemolithotrophic microorganisms, oxidizing sulphides to elemental sulphur [21]. Elemental sulphur is usually stored in internal small vesicles giving the mats a white appearance. In a second step, when sulphur lacks in the environment, it is further oxidized to sulphate [22]. The sulphur-oxidizing metabolism can be determined using different approaches. In culture, sulphur-oxidizing bacteria can grow in presence of sulphur [11]. A molecular approach identifying the genes involved in sulphur oxidation pathways (*i*.*e*. *apr* or *sox* genes) [23,24] or measurements of the negative δ^13^C content proving *Beggiatoa* chemoautotrophy [25,26] can also be performed. Moreover, Energy-Dispersive X-ray (EDX) and Raman spectroscopy have already been used in a few studies in order to detect the elemental sulphur stored in the sulphur-oxidizing microorganisms [16,18,27]. These autotrophic bacteria require CO_2_ for growth, but can also use acetate as a carbon source [28]. Furthermore, *Beggiatoa* species are involved in the nitrogen cycle, the large vacuolated species being capable of nitrate respiration [29], whereas non-vacuolated species can use both nitrate and nitrite as nitrogen sources [30]. Recently, some species have also been identified as diazotrophs [31]. Thus, their contribution to the sulphur, carbon and nitrogen biogeochemical cycles allows these species to recycle the chemical elements and provide food for heterotrophic organisms [32].

Here, we describe the major bacteria forming a marine mangrove white mat in Guadeloupe (French West Indies) and identify them as two new species of large filamentous sulphur-oxidizing affiliating with the family Beggiatoaceae. Phylogenetic analysis based on 16S rDNA gene, ultrastructural and biochemical analyses as well as *in situ* hybridization were conducted to identify the new organisms. Lipid analysis was also carried out to support the phylogeny. EDX cartography was performed to assess the autotrophic character of these species. Finally, we characterized the chemical environment of the mat, performing measurements of sulphides and oxygen rates under mesocosm conditions.

## Materials and Methods

### Sampling

Colorless filaments were collected in marine mangrove of Guadeloupe (French West Indies) at 16°N, 61.5°W. They were sampled with 60 mL syringe and placed in large glass Petri dishes once back to the lab in order to select the filaments under a dissecting microscope. No specific permissions were required from these locations and activities. Our study did not involve endangered or protected species.

### Fluorescence *in situ* Hybridization

Colorless filaments were prepared for FISH analysis according to previously described protocols [13]. After hybridization, samples were observed in MilliQ water with a drop of Vectashield using an epifluorescence Nikon microscope Eclipse 80i.

FISH analyses were performed using universal probes for Bacteria (EUB 338) [33], NON338 [34], as negative control and BEG572 (5’-CAACCGCCTACGTACGCT-3’) and BEG282 (5’-GGATTGCTGTCTTGGTAAGC-3’) for morphotype 1 and morphotype 2, respectively, that were specifically designed from the 16S rDNA bacterial sequences obtained in this study.

The specific probes (labelled with Cy3) were designed manually. Probes 16S ss-rRNA localization was optimized according to Fuchs *et al*. [35]. The probe’s specificity was further tested with the online Probes Match tool provided by the Ribosomal Database Project [36].

### Ultrastructural analysis

The ultrastructure of the colorless filaments was determined using a Scanning Electron Microscope (SEM Quanta 250, FEI). To this end, the bacterial filaments were fixed at 4°C in 2.5% glutaraldehyde in 0.1M cacodylate buffer (pH 7.2) which was made iso-osmotic to sea water by addition of sodium chloride and calcium chloride. Samples were then kept at 4°C until analysis. For conventional SEM analysis, samples were briefly rinsed, then dehydrated through a graded acetone series before drying with CO_2_ using a critical point drier machine (EM CPD300, Leica). The samples were then sputter-coated with gold (Sputter Coater SC500, Biorad).

For EDX analysis, in order to avoid salt crystallization, samples were rinsed three times with deionized water, before observation with an ESEM Quanta 250 (FEI) operating from 10 to 20 kV under an environmental pressure of 7 Torrs at 5°C. EDX spectra were obtained using a M-max 500 mm^2^ Oxford detector.

For Transmission Electron Microscopy (TEM) analysis, prefixed bacterial filaments were washed twice in 0.1M sodium cacodylate buffer in order to remove aldehydes before fixation for 45 min at room temperature in 1% osmium tetroxide in the same buffer. Then, samples were rinsed in distilled water, and post-fixed with 2% aqueous uranyl acetate for one hour more. After a rinse in distilled water, each sample was dehydrated through a graded acetone series and embedded in Epon-Araldite [37]. Thin sections (60 nm thick) were contrasted 30 min in 2% aqueous uranyl acetate and 10 min in 0.1% lead citrate before examination in a TEM LeO 912.

### DNA extraction and PCR amplification

DNA was extracted from colorless filaments using DNeasy Blood & Tissue kit (Qiagen) according to the manufacturer’s instructions. 16S rDNA were amplified using primers 8F/ 907R (for morphotype 1) and 8F/1492R (for morphotype 2) as previously described [38, 39]. PCR amplifications were performed as follows: 95°C for 5 min, 35 cycles of 94°C 30 s, 58°C 45 s, 72°C 1min 30 sec and finally 72°C 7 min. PCR products were purified using QIAquick PCR purification Kit (Qiagen) and cloned with pGEM-T cloning kit (Promega) according to manufacturer’s instructions. Inserts from 20 positive clones of each construction were fully sequenced by Genoscreen (http://www.genoscreen.com) using vector primers T7 and SP6. The sequences obtained in this study were deposited in the GenBank database under accession no. KF892059 and KF892060.

### Phylogenetic analysis

The 16S rDNA gene sequences obtained were compared with the National Center of Biotechnology information (NCBI) (http://www.ncbi.nlm.nih.gov) database using BLAST [40]. Best hits were included in phylogenetic analyses. The phylogenetic analyses were conducted using MEGA version 5 [41]. Sequences were aligned using SINA alignment service [42] of the SILVA web site (http://www.arb-silva.de) and alignments were checked manually. The phylogenetic tree was constructed from the multiple-aligned data using the Neighbor Joining (NJ) method with Tamurai-Nei as genetic distance model. Nodes robustness was assessed by performing 1000 bootstrap replicates, and only bootstrap values above 49% are indicated at the nodes of the tree. *Leucothrix mucor*, *Thiothrix nivea*, and *Achromatium spp* were used as outgroup.

### Nomenclature

The electronic version of this article in Portable Document Format (PDF) in a work with an ISSN or ISBN will represent a published work according to the International Code of Nomenclature for algae, fungi, and plants, and hence the new names contained in the electronic publication of a PLOS ONE article are effectively published under that Code from the electronic edition alone, so there is no longer any need to provide printed copies.

The online version of this work is archived and available from the following digital repositories: PubMed Central, LOCKSS.

### Lipid characterization

Lipids were extracted from freeze-dried biomass of the two morphotypes using a modified Bligh and Dyer extraction [43]. The extracts were subjected to acidic methanolysis (ibid.) in order to remove polar head groups and to obtain free fatty acids. An aliquot was methylated with BF_3_-MeOH, treated with BSTFA in pyridine and subsequently analyzed by gas chromatography-mass spectrometry (GC-MS) using a TRACE GC with a DSQ-MS, using a fused silica capillary column (25 m, 0.32 mm internal diameter) coated with CP Sil-5 (film thickness 0.12 μm) and helium as a carrier gas. To determine the double bond position of the fatty acids, they were derivatized with dimethyldisulfide/I_2_ and the resulting methyltioethers were analysed by GC/MS.

### Sulphide measurements

***In situ* measurements**. In an attempt to characterize the *in situ* conditions, 10 measurements were performed in sediment areas covered by the white bacterial mat with autonomous potentiometric captors. Sulphide and pH captors were both used in order to calculate the sulphide rates. The sulphide and the pH measuring system were the same as the one previously described [12] and have been used in various habitats [12,44]. The electrodes were calibrated in the laboratory before deployment.

A series of 10 short term measurements was performed in 1 cm sediment under several patches of white mat with tightly attached sulphide and pH electrodes. The average of these measurements was calculated with standard deviation.

**Mesocosm measurements**. Mangrove sediment was brought to the laboratory and installed in a glass recipient until the sediment was reorganized. Mat was collected from the field the day after and transferred immediately (within 1 hour) into the mesocosm on the sediment (see S1 Fig.).

Oxygen and sulphide profile measurements were carried out using Clark-style oxygen (Oxy100) and sulphide (H_2_S100) microsensors with a 10μm tip manufactured by Unisense (Aarhus, Denmark) connected to a four channel Unisense picoammeter. Calibrations were performed according to Unisense instructions. The pH was measured with autonomous probe similar to the one described [44] fixed to the micromanipulator.

Vertical profiles were determined by moving the microelectrodes using a micromanipulator into the mat and recording the electrical current with SensorBasic software. Total sulphide concentrations (S^2-^_tot_ = H_2_S+HS^-^+S^2-^) were calculated taking into account the measured pH and salinity [45] using a pK = 6.51.

## Results

### Morphology

**Fig 1:**
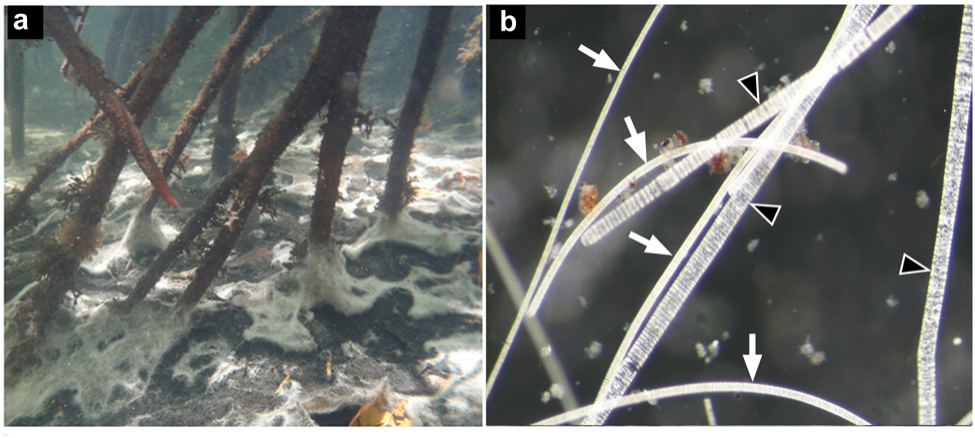
Photographs of the *Beggiatoa* mat. Underwater picture of patches of white mat on the mangrove sediment (a). Sample of colorless filaments observed under dissecting microscope (b): Two morphotypes are visible; black arrowheads indicate morphotype 1, and white arrows indicate morphotype 2.

The large colorless filamentous microorganisms were collected from extensive white mats (Fig. 1a) located near the *Rhizophora mangle* roots in the tropical mangrove swamp in Guadeloupe. By light microscopy, it was noticed that the mat was composed mainly of 2 morphotypes with large colorless filaments (Fig. 1b).

**Fig 2:**
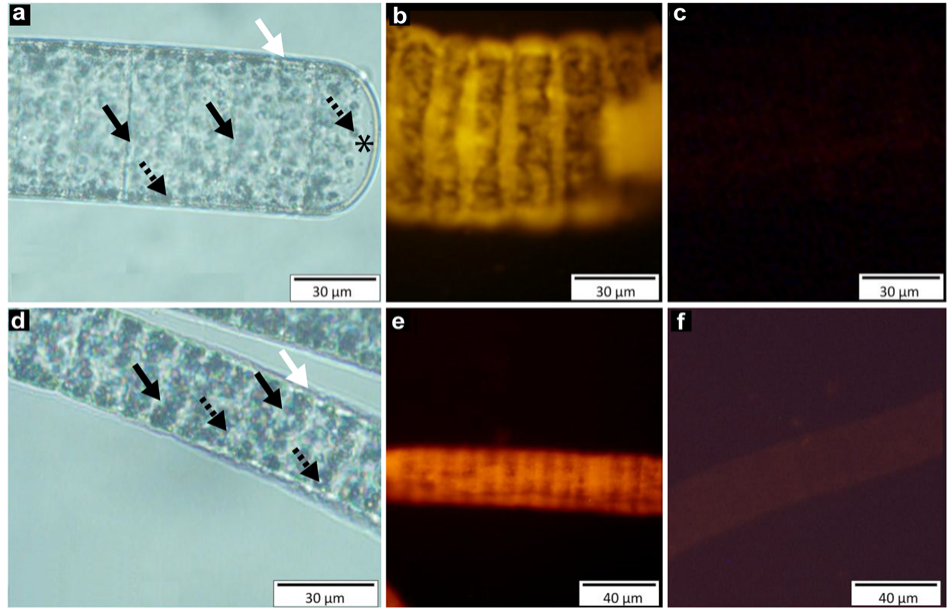
Structure and identification of the two *Beggiatoa* morphotypes. Light microphotographs of morphotype 1 (a) and morphotype 2 (d), respectively. White arrows highlight the white sheath, the black arrows point out the membranes separating two bacterial cells, and the dotted arrows highlight the sulphur vesicles. The apex of the morphotype 1 filament is marked by a black star. The right identification of the two morphotypes is confirmed by the positive hybridization with the specific probes (BEG572F for morphotype 1 and BEG282F for morphotype 2) designed from each bacterial sequence obtained in this study (b and e are morphotypes 1 and 2, respectively). NONEUB probe was used as negative control (c and f for morphotypes 1 and 2, respectively).

**Fig 3:**
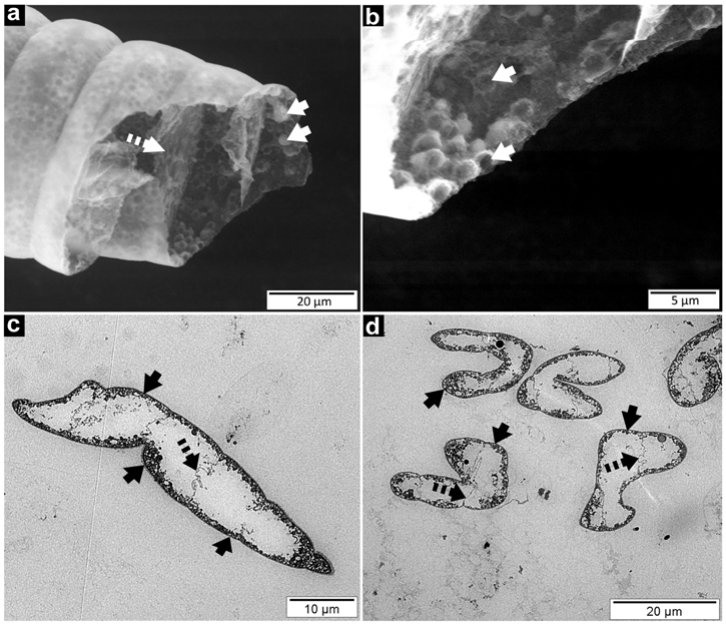
Ultrastructure of the two *Beggiatoa* morphotypes. SEM microphotographs of morphotype 1 (a-b). These images highlighted small vesicles (white arrows) absent from the membranes separating two adjacent cells (dotted arrows). On higher magnification (b), some of these vesicles are fractured (white arrows), and appeared linked to the membranes. TEM microphotographs of the morphotype 1 (c) and morphotype 2 (d) highlight a large central empty space with all the cytoplasmic content postponed on the external membranes. The small vesicles (black arrows) also appear empty due to the loss of sulphur during dehydration process. They are absent from the membranes (dotted arrows) separating two adjacent cells.

The two colorless morphotypes described here are assemblages of cells constituting filaments of a total length of up to 30 mm (Fig. 2 a, d.). Light microscopy observations showed that the filaments of morphotype 1 (Fig. 2a-b; Fig. 3a) are an assemblage of discoid cells of 60 μm wide and 18.6 μm long, whereas the filaments of morphotype 2 (Fig. 2d) are a chain of cylindrical smaller cells, up to 30 μm wide and 3.8 μm long. In both filaments, small vesicles can be observed in the cells and the external membrane appeared thicker due to the presence of a thin sheath which can be removed by critical point treatment in SEM observations.

**Fig 4:**
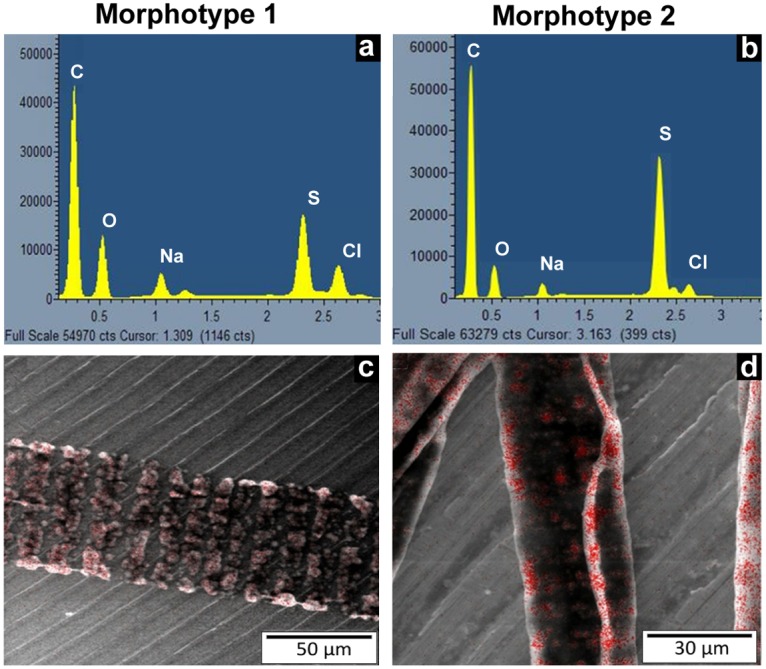
Sulphide metabolism of the *Beggiatoa spp*. The EDX spectra of morphotypes 1 (a) and 2 (b) obtained from non-dehydrated samples observed under an ESEM showed that the bacteria contain elemental sulphur. The sulphur mapping localizes this element (marked in red) inside the cells within the cytoplasmic granules (c and d).

SEM (Fig. 3a) and TEM (Fig. 3c-d) observations showed that no external bacteria are encountered on the filament. The small vesicles visible under a light microscope (Fig. 2a-b) appeared, according to SEM observations, as pasted to the cell inner membranes. However, none of these vesicles were observed on the membranes separating two adjacent cells within the same filament (Fig. 3a). This observation was confirmed by TEM sections, which showed that these vesicles were linked to the inner membrane but not merged with it (Fig. 3c). No free vesicles were observed in the cell cytoplasm, whatever section orientation was used (Fig. 3c-d). Because sulphur is dissolved during dehydration processes, these empty vesicles observed in SEM fracture (Fig. 3b) and TEM sections (Fig. 3c-d) could be identified as sulphur vesicles. Their identification was performed with EDX analysis (Fig. 4). The entire bacterial content appeared projected over the outside of the cell, attached to the inner membranes, releasing a central space not bounded by a membrane.

### Phylogenetic analysis

**Fig 5:**
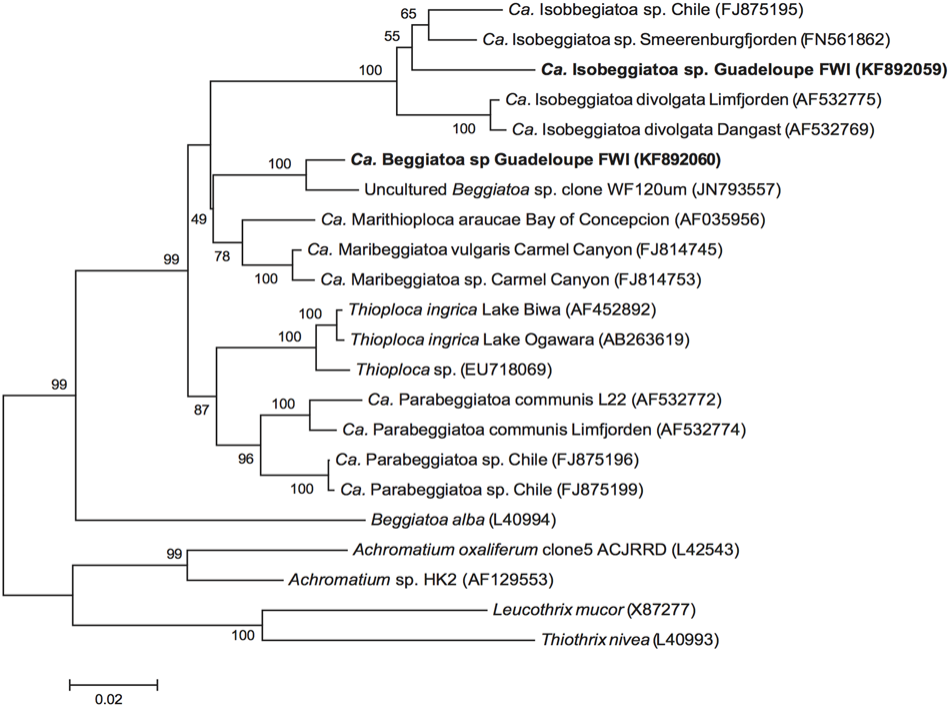
Neighbor joining (NJ) tree displaying the phylogenetic relationships between the *Candidatus* Beggiatoa sp. Guadeloupe FWI and *Candidatus* Isobeggiatoa sp. Guadeloupe FWI (in bold) with other colorless large sulphur bacteria. Phylogenetic tree based on the analysis of 16S rDNA gene sequences of 925 nucleotides. Node robustness was assessed by performing 1000 bootstrap replicates. Only bootstrap values more than 49% are shown at each node. *Leucothrix mucor*, *Thiothrix nivea*, and *Achromatium spp* were used as outgroup. The scale bar corresponds to 0.02 changes per nucleotide.

The phylogenetic analysis was performed accordingly to the modern classification of large sulphur bacteria [41]. Neighbor-Joining (NJ) tree based on partial 16S rDNA sequences (925bp) revealed that the morphotype 1 forms a distinct clade with Uncultured *Beggiatoa sp*. clone WF120μm (Fig. 5) which falls into *Candidatus* Maribeggiatoa group [5]. The sister group was supported by the robust branch of the phylogenetic tree (100% bootstrap support from 1000 replicates). In contrast, phylogenetic analysis identified morphotype 2 as a sister group of *Candidatus* Isobeggiatoa spp. Thus, we proposed to name morphotype 1 strain as *Candidatus* Beggiatoa sp. Guadeloupe FWI and morphotype 2 strain as *Candidatus* Isobeggiatoa sp. Guadeloupe FWI in reference to the sister group they belong to and to the sampling site: Guadeloupe French West Indies.

The phylogenetic relationship of these two species was checked by *in situ* hybridization using specific probes (BEG572F for morphotype 1 and BEG282F for morphotype 2) designed from each bacterial sequence obtained in this study (Fig. 2b, e). The positive hybridization shown in Fig. 2b, e shows that the two morphotypes observed in the white mat correspond to the phylogenetic sequences previously obtained. A negative control was performed using NON338 probe (Fig. 2c, f). Lipid analysis showed that the fatty acids mainly consisted of C_16_ and C_18_ fatty acids with 0–1 double bonds and minor amounts of C_20_ fatty acids in both morphotypes, with morphotype 2 containing significant amounts of a C_20_ polyunsaturated fatty acid (Table 1). The double bond position in the C_18:1_ fatty acid in morphotype 2 was determined by DMDS adduction as ω-7, and, while concentrations of C_16:1_ and C_18:1_ fatty acids in morphotype 1 were too low for analysis after derivatization, retention times indicate an ω-7 position for those too.

**Table 1:** Distribution of fatty acid methyl esters (FAME) in both morphotypes in % as determined by GC-MS.

ORGANISMS	FAME	%
Morphotype 1	C16:1	6.6
	C16:0	19.3
	C18:1	19.8
	C18:0	50.7
	C20:0	3.6
Morphotype 2	C16:1	7.5
	C16:0	26.5
	C18:1	51.1
	C18:0	7.7
	C20:pufa	7.2

Numbers indicate carbon numbers and number of double bonds if any, as well as double bond position if determined. pufa = polyunsaturated fatty acid.

### Sulphur-oxidizing metabolism

In our study, EDXS analysis was performed using an environmental SEM (ESEM) allowing the observation of fully hydrated biological samples, and thus elemental sulphur was not dissolved during the preparation process of the samples. The EDX spectra showed that sulphur is the main element present within the organisms (Fig. 4a, b). Moreover, EDX cartography allowed to localize the elemental sulphur within granules that appeared as empty vesicles according to conventional SEM (Fig. 3b) and TEM pictures (Fig. 3c, d). Thus, both structural and EDXS analyses demonstrate that the vesicles observed by light and electron microscope are sulphur storage granules (Fig. 2a, d; Fig. 3a-b).

### Sulphide measurements

**Fig 6:**
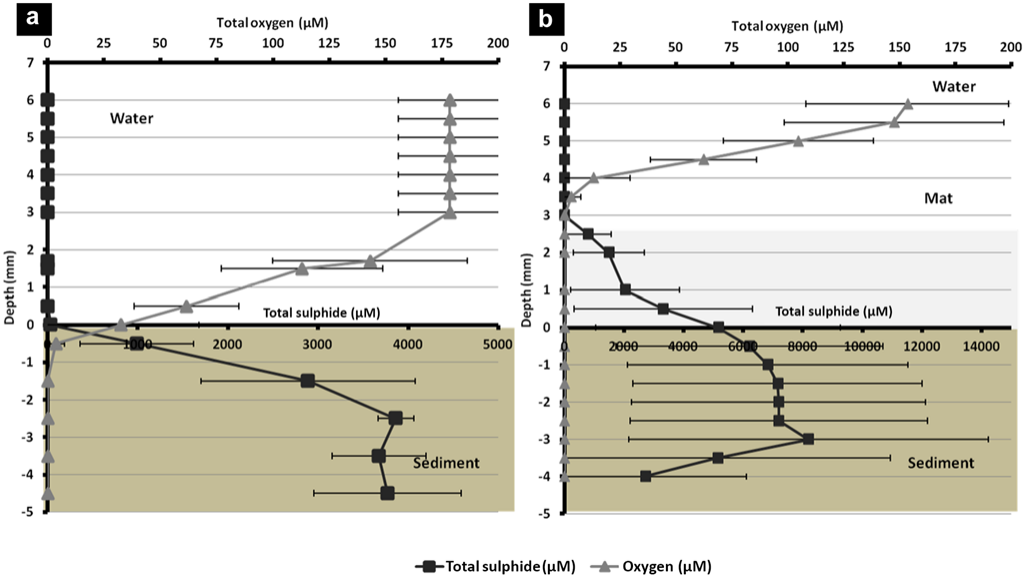
Vertical distribution of sulphide and oxygen in mangrove sediment under mesocosm conditions. Representative (square) total sulphide and (triangle) oxygen microgradients measured into the sediment on the mesocosm system (a) without mat and (b) with mat. Concentrations are expressed in μM. Error bars = one standard deviation of the mean.

In order to characterize the mat environment in its natural biotope, *in situ* sulphide measurements were performed. The values obtained in mangrove from the ten profiles ranged from 189 μM to 2396 μM, with an average of 1187 μM (±728). Profiles from mesocosm experiments in the laboratory, in presence or absence of a bacterial mat, are shown in Fig. 6. In absence of bacterial mats (Fig. 6a), oxygen penetrated 0.5 mm into the sediment while sulphides were detected (detection level ~1 *μ* m) below a depth of 0.2 mm. Sulphide concentrations reached 988 μM (± 627) at 0.5 mm depth and increased continuously with depth. Therefore, the anaerobic sulphate-reducing bacteria (SBR) contained in the sediment were functional and produced sulphides by degradation of the organic matter by sulphate reduction.

In contrast, when the bacterial mat was present on the sediment (Fig. 6b), the oxygen concentration in the water column gradually decreased from 153 μM (± 45) to zero 3 mm above the sediment while sulphides were detected before entering the sediment. It was also observed that sulphide rates increased with depth until 3 mm with a maximum of 8197 μM (± 6030) and then decreased.

In the presence of the mat, a slope rupture of the sulphide concentration curve can be noticed, which means that the sulphide concentrations decreased quicker in the mat than in the sediment while it diffused to the surface. This data suggests that the bacterial mat consumed the sulphides coming from the sediment (due to SBR activity) quicker than natural sulphide oxidation by oxygen present in the seawater.

## Discussion

### Phylogenetic placement of the new Beggiatoaceae species

In this study we described two new species of Beggiatoaceae family, which are forming microbial mats in marine mangroves from the Caribbean. According to the recently updated large sulphur bacteria phylogeny by Salman *et al* [46,47] our sequences studied here belong to two distinct taxa, *Candidatus* Isobeggiatoa sp. and *Candidatus* Maribeggiatoa sp., and share many characteristics with *Beggiatoa alba*, the type specie of Beggiatoaceae. In fact, for the two bacteria described here, the same morphology can be observed: multicellular filaments harbouring discoid cells with sulphur granules visible into incident light. Furthermore, both filaments can move by gliding on solid surface and possess a sheath.

Morphotype 1 is phylogenetically close to another bacterial species of 120 μm diameter identified as *Maribeggiatoa* [5] suggesting a division based on cell diameter within the *Maribeggiatoa* genus: one cluster harbouring species with a diameter between 12 to 18 μm, and a second one with diameter between 25 to 37 μm. Our data suggest that morphotype 1 and Uncultured *Beggiatoa* sp. clone 120 μm reported by Mckay *et al*. could form a third cluster with diameters ranging from 60 μm to 120 μm [5]. Additional phylotypes and new taxa identification are needed in order to clarify this cluster. In contrast, morphotype 2 clearly belongs to *Isobeggiatoa* group, which only gathers filaments with diameters between 10 and 40 μm. Interestingly, the sequence obtained here only shared 94% identity with available sequences from Genbank. According to some authors, percentages lower than 95% could indicate a new genus [48,49]. Furthermore, all the *Isobeggiatoa* spp. sequences available are from cold temperate water species (Denmark and Germany), and Antarctic environments [46].

The results of the lipid analysis (Table 1) confirmed the genetic results, as the main fatty acids detected contained 16 and 18 carbon atoms with 0–1 ω-7 double bonds, concurrent with previously published results for *Beggiatoa* [50,51] and other sulphur-oxidizing bacteria *Thioploca* and *Thiomargarita* [51,52]. Interestingly, Jacq and co-authors [53], who characterized two types of filamentous bacteria retrieved from subtidal hydrothermal vents in southern California, were the only ones to also report small, but significant amounts of polyunsaturated C_20_ fatty acids in both phenotypes. A C_20_ polyunsaturated fatty acid was only detected in morphotype 2, phylogenetically characterized here as *Isobeggiatoa*, but was absent in morphotype 1 (i.e. *Beggiatoa*), suggesting that the *Beggiatoa*-like mats observed in subtidal hydrothermal vents may have been *Isobeggiatoa* [53]. C_15_ and C_17_ fatty acids, which are characteristic for sulphate-reducing bacteria, were absent.

This result shows that information based on 16S rRNA gene sequences is insufficient to identify new species and how it is necessary to use multiphasic approach to classify them. Nevertheless, further molecular investigations involving additional marker genes (i.e. 23S rDNA, ITS) and other multiphasic approach (e.g., physiological traits) could be used in order to resolve in depth the phylogeny of these species [46].

### Sulphur metabolism of the new Beggiatoa species

*Beggiatoa* mats, as all microbial mats, are self-sustaining communities that support all major biogeochemical cycles [54]. The characterization of their chemical environments, either *in situ* or in mesocosms, by sensor measurements can provide information about their contributions to the ecosystem [55,56,57]. Mesocosm measurements were similar to those observed in previous studies undertaken in marine mangroves. Sulphide concentration increased with depth in the sediment in absence of mat [12,58]. Moreover, in our study, under the *Beggiatoa* mat, a decrease of sulphide concentration was observed after 4 mm depth. This phenomenon was already noticed in an ultramafic hydrothermal vent field [59], and in a sulfidic cave [60] where the sulphide rate did not only increase with depth, as shown in numerous studies [19,61]. No explanation for this phenomenon was given in the hydrothermal vent. However, Macalady *et al*. showed that in the sulfidic caves, it could be explained by diffusion-controlled transport and also by the fact that in sulfidic caves, sulphides diffused both from water above and from sediment below [60].

Measurements in a mangrove under a *Beggiatoa* mat showed that oxygen was fully consumed 2 cm above the mat [19]. In our study, an anoxic zone was present a few mm above the mat. Furthermore, under the filament network, sulphide concentrations were more important in the mesocosms than previously reported in literature. Indeed, a concentration of 1489 μM (± 1328) of sulphide was reached at 2 mm depth into the sediment in mesocosm, whereas in our *in situ* measurements, at 1 cm, we measured an average concentration of sulphide of 1193 μM (±728), similar to the measurements performed in the Twin Cays mangrove, where 1400 μM (± 1000) is reached at 1 cm depth [19]. Although concentrations are higher in mesocosm at this depth, at 1 cm, they are lower in the mesocosm than *in situ*. A significant heterogeneity existed within the sediment, *in situ*, and in the mesocosm as evident by the large standard deviations. This heterogeneity allowed us, to date, to consider the mesocosm as similar to *in situ* conditions. To our knowledge, no study has been conducted in mangrove habitats on *Beggiatoa* mats using microprobes. The concentrations measured by Lee *et al*. were obtained by colorimetry [19], with less precision than the measurements performed here using probes.

In two previous studies done in mangrove environments, sulphide concentrations were always lower than 1000 μM for 10 cm depth [12,58]. However, the locations were not next to or under *Beggiatoa* mats which are expected to be present at places where higher sulphide concentrations are available. Indeed, members of the Beggiatoaceae family are known to migrate in order to find the best gradient sulphur/oxygen for their development [62,63]. High currents or other mechanisms could also explain the mats’ localization into the mangroves.

Our results are similar to those obtained in other environments. In hydrothermal vents and in the Santa Barbara Basin, it was shown that all oxygen was consumed within the first millimeters of the sediment while sulphide concentrations increased with depth [59,64]. However, in these two environments the sulphide concentrations were 25 to 150 times lower than those observed during our mesocosm experiment. In the hydrothermal vents, the maximum of sulphide concentration observed was 250 μM at 30 mm sediment depth [59], and in the Santa Barbara basin, a maximum of 50 μM was observed at 12 mm sediment depth [64].

The morphological study of *Candidatus* Beggiatoa sp. Guadeloupe FWI and *Candidatus* Isobeggiatoa sp. Guadeloupe FWI, highlighted that the sulphur inclusions visible in SEM and TEM images and identified by EDX, are joined to the plasmic membrane and are absent from membranes separating two adjacent cells. However, it was impossible to distinguish whether the sulphur granules were surrounded by a single membrane against the outer membrane, in invaginations of the cytoplasmic membrane, as previously suggested [28].

The *Beggiatoa* species described here are the predominant species in the filament network and thus probably the main microorganisms responsible for the sulphur consumption observed in the mat. Nevertheless, other sulphur-oxidizing bacteria could also participate in sulphide oxidation. The presence and activity of bacteria other than the giant *Beggiatoa* spp. were not determined in this study. Mesocosm measurements showed that, while oxygen was absent from the first millimeter of the mat, *Beggiatoa* cells were still present. These were probably cells from the anaerobic layer using dissimilatory nitrate reduction to ammonium in order to oxidize sulphur. Their need to oxidize sulphur and/or ammonia would cause migration to the oxic sediment layer. Indeed, SEM and TEM images highlighted a large free space in the cell with all the cytoplasmic content positioned against the outer membrane of the cell. These large vacuoles could be the nitrate vacuoles already encountered in previous large marine *Beggiatoa spp*. [27,29,63,65,66], and observed in *Isobeggiatoa* and *Marithioploca* strains [41]. These nitrate vacuoles allow the bacteria to survive anaerobically, oxidizing sulphides through nitrate reduction into dihydrogen and ammonia [1,30,67,68,69].

In our study, the internal component of the central space was not identified but TEM images showed that the empty area has no intracytoplasmic membrane. This is in accordance with De Albuquerque *et al*., who showed that the vacuoles have no internal membranes into marine and hypersalines studied mats [27], as observed also in *Thioploca* [69]. However, some marine sulphur-oxidizing bacteria from *Thiothrix* genera showed such vacuoles with no nitrate accumulation [70]. Thus, in absence of more information about the nature of the vacuoles and the nitrification rates of the mat, it is impossible to draw conclusions on the metabolism of nitrogen in these two new species of Beggiatoaceae.

It could be interesting to study the ammonium consumption of the *Beggiatoa* mat in marine mangrove in order to estimate their contribution to the nitrogen cycle regarding the mat composition. Furthermore, a recent study has shown that some non-marine *Beggiatoa* spp. from sulfidic caves are able to fix nitrogen [31]. This suggests that is possible that also *Candidatus* Beggiatoa sp. Guadeloupe FWI and *Candidatus* Isobeggiatoa sp. Guadeloupe FWI could fix nitrogen.

The *Beggiatoa* mats are also known to provide food for benthic foraminifera in temperate tidal flats and Antarctic shallow waters [64], but also for meiofauna and macrofauna of the Denmark cold waters [61]. In mangroves, the interactions between meiofauna and microbial mats have shown that some nematods and annelids feed on these mats, so the mat could be the source of a complex food web [71]. Thus, a detailed study of the interactions between these compartments will help to understand how the *Beggiatoa* mats contribute to the mangrove ecosystem.

This multidisciplinary study has revealed two new species of *Maribeggiatoa* and *Isobeggiatoa*, inhabiting the marine mangrove. This study is the first evidence for the presence of *Isobeggiatoa* spp. outside of northern Europe or Arctic waters. The multiphasic approach with use of microprobes, electron microscopy, lipid and phylogenetic analysis, has provided detailed information on species, and their sulphidic environment. Furthermore, the mesocosm study addresses some issues of the metabolism of these two species; and the results indicate that the role of the central vacuole is related to the dissimilatory nitrate reduction to ammonium. Our results are a first approach to ultimately understand the contribution of Beggiatoaceae-dominated microbial mats to the biochemical cycles and food web of mangroves. They could constitute a base for further studies dealing with marine mangrove microbial mats.

## Supporting Information

S1 Fig Micromanipulator for measurements with microsensors in mesocosm. (TIF)
