# Supplementary figures and images for: Two New Beggiatoa Species Inhabiting Marine Mangrove Sediments in the Caribbean
Source: PLoS One. 2015 Feb 17;10(2):e0117832. doi: 10.1371/journal.pone.0117832 (PMC4331518; doi:10.1371/journal.pone.0117832)

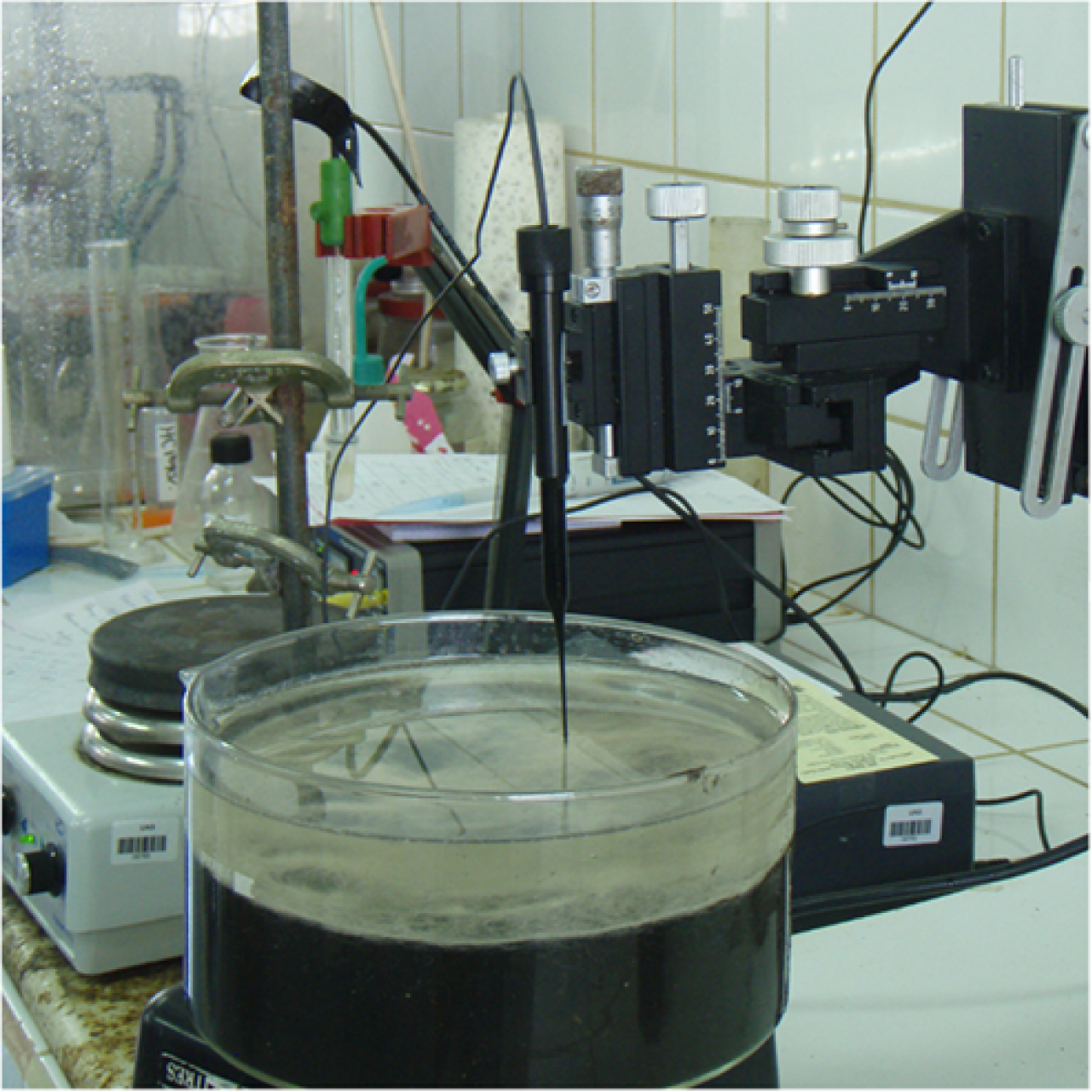

Supplement: S1 Fig — (TIF) [file pone.0117832.s001.tif]
